# Supplementary material for: The Systems Biology Research Tool: evolvable open-source software
Source: BMC Syst Biol. 2008 Jun 29;2:55. doi: 10.1186/1752-0509-2-55 (PMC2446383; doi:10.1186/1752-0509-2-55)
Supplement: Additional file 1 — SBRT Archive. An archive of the current version of the Systems Biology Research Tool. [file 1752-0509-2-55-S1.zip › sbrt-1.4.0/doc/developers_guide/api/sbrt/shell/proc/fba/BiggBracketParserV1.html]

BiggBracketParserV1


---


|  |  |  |
| --- | --- | --- |
| **Overview** \| **Package** \| **Class** \| **Use** \| **Tree** \| **Deprecated** \| **Index** \| **Help** | |  |
| PREV CLASS   **NEXT CLASS** | **FRAMES**    **NO FRAMES**     **All Classes** |  |
| SUMMARY: NESTED \| FIELD \| CONSTR \| METHOD | DETAIL: FIELD \| CONSTR \| METHOD |  |


---


## sbrt.shell.proc.fba Class BiggBracketParserV1

```
java.lang.Object
  sbrt.shell.proc.fba.BiggBracketParserV1
```

---

``` public class BiggBracketParserV1 extends java.lang.Object ```

This class is used to parse bracketed expressions contained in SBML files
from the BiGG Database. These expressions are used to represent
gene-protein-reaction associations using boolean logic. An example would be:

((b3951 and b3952) or b3114 or (b0902 and b0903)).

All provided expressions are assumed to be formatted like the above example
and free of errors. If this is not the case, the results of parsing are
unpredictable.

**Author:**
:   This class was written and documented by Jeremiah Wright while in the
    Wagner lab.

---

**Constructor Summary**

`BiggBracketParserV1()`


| **Method Summary** | |
| --- | --- |
| `static void` | `main(java.lang.String[] args)`             Used for testing purposes. |
| `Or<And<java.lang.String>>` | `parse(java.lang.String string)`             Parses the provided BiGG style bracketed expression. |

**Methods inherited from class java.lang.Object**

`clone, equals, finalize, getClass, hashCode, notify, notifyAll, toString, wait, wait, wait`

**Constructor Detail**

### BiggBracketParserV1

```
public BiggBracketParserV1()
```


**Method Detail**

### main

```
public static void main(java.lang.String[] args)
```

:   Used for testing purposes.

    :   **Parameters:**: `args` - ignored

---


### parse

```
public Or<And<java.lang.String>> parse(java.lang.String string)
```

:   Parses the provided BiGG style bracketed expression.

    :   **Parameters:**: `string` - a BiGG-type bracketed expressions with parantheses used as brackets. **Returns:**: the boolean rules represented by the string.


---


|  |  |  |
| --- | --- | --- |
| **Overview** \| **Package** \| **Class** \| **Use** \| **Tree** \| **Deprecated** \| **Index** \| **Help** | |  |
| PREV CLASS   **NEXT CLASS** | **FRAMES**    **NO FRAMES**     **All Classes** |  |
| SUMMARY: NESTED \| FIELD \| CONSTR \| METHOD | DETAIL: FIELD \| CONSTR \| METHOD |  |


---
